# Enhancing patient positioning accuracy: evaluating daily cone beam computed tomography in the halcyon system

**DOI:** 10.1093/jjco/hyaf067

**Published:** 2025-04-23

**Authors:** Duong Thanh Tai, Luong Tien Phat, Tran Trung Kien, Nguyen Ngoc Anh, Nguyen Xuan Hai, Peter Sandwall, David Bradley, James C L Chow

**Affiliations:** Department of Medical Physics, Faculty of Medicine, Nguyen Tat Thanh University, 298-300A Nguyen Tat Thanh Street, Ward 13, District 4, Ho Chi Minh City 700000, Vietnam; Department of Radiation Oncology, University Medical Shing Mark Hospital, 1054 QL51, Long Binh Tan Ward, Bien Hoa City, Dong Nai 810000, Vietnam; Phenikaa Institute for Advanced Study (PIAS), Phenikaa University, 01 Nguyen Trac, Ha Dong District, Hanoi 12116, Vietnam; Phenikaa Institute for Advanced Study (PIAS), Phenikaa University, 01 Nguyen Trac, Ha Dong District, Hanoi 12116, Vietnam; Center for Analytical Techniques, Dalat Nuclear Research Institute, 01 Nguyen Tu Luc Street, Ward 8, Da Lat City, Lam Dong Province 670000, Vietnam; Department of Radiation Oncology, OhioHealth Mansfield Hospital, 330 Glessner Avenue, Mansfield, OH 44903, United States; Applied Radiation Physics and Technologies Group, Department of Engineering, Sunway University, No. 5 Jalan Universiti, Bandar Sunway, Subang Jaya, 46150 Petaling Jaya, Selangor, Malaysia; School of Mathematics and Physics, University of Surrey, Stag Hill Campus, University Road, Guildford, Surrey GU2 7XH, United Kingdom; Department of Radiation Oncology, University of Toronto, 610 University Avenue, Toronto, Ontario M5G 2M9, Canada; Radiation Medicine Program, Princess Margaret Cancer Centre, University Health Network, 610 University Avenue, Toronto, Ontario M5G 1X6, Canada

**Keywords:** halcyon, patient setup error, cone beam computed tomography, patient positioning, radiation therapy, VMAT, IMRT

## Abstract

**Background:**

Precise patient positioning is crucial for successful radiotherapy, ensuring accurate delivery of radiation to tumors while minimizing exposure to healthy tissues. Positional errors can significantly impact treatment efficacy and increase side effects. This study evaluates the effectiveness of daily cone beam computed tomography (CBCT) imaging in the Halcyon system for detecting and correcting patient misalignments across various cancer types and treatment sites.

**Methods:**

A retrospective analysis was conducted on 411 patients treated with the Varian Halcyon linear accelerator from August 2022 to August 2024. Patients were grouped based on tumor location: Head and Neck (118 patients), Chest (188 patients), and Pelvis (105 patients). Daily pre-treatment CBCT scans were performed to verify positioning, with shifts in the *x*, *y*, and *z* axes quantified and adjusted using automated couch corrections.

**Results:**

The study revealed average positional shifts along the *x*-axis of ~0.112 cm, while both the Chest and Pelvic groups recorded 0.194 cm. The *y*-axis deviations were 0.135 cm for Head and Neck, 0.206 cm for Chest, and 0.195 cm for the Pelvis. On the *z*-axis, a mean deviation of 0.07 cm was found for the Head and Neck group, while 0.11 cm for the Chest group, and 0.085 cm for the Pelvic group. The Head and Neck group exhibited the smallest standard deviations across all axes, indicating greater positional consistency. Normalized density distributions showed distinct emergent patterns, the Head and Neck group showing tighter distributions compared to the broader distributions observed in the Chest and Pelvic groups.

**Conclusions:**

Daily CBCT imaging in the Halcyon system significantly enhances patient positioning accuracy in radiotherapy. The findings demonstrate that this approach minimizes positional shifts, particularly in the Head and Neck region, essential for optimizing treatment outcomes and reducing the risk of adverse effects. Future studies should further explore the integration of advanced imaging techniques to improve precision in patient positioning.

## Teaser

Daily cone beam computed tomography imaging improves radiotherapy precision, reducing positional shifts, and ensuring optimal tumor targeting in Halcyon system across multiple cancer treatment sites.

## Introduction

Precise patient positioning is fundamental to successful radiotherapy, ensuring therapeutic radiation doses are accurately directed to the tumor, while simultaneously minimizing the risk of unnecessary exposure to surrounding healthy tissues. Misalignments during treatment are well-documented contributing factors to suboptimal therapeutic outcomes, increasing the likelihood of side effects and reducing treatment efficacy [1–4]. One of the primary challenges in radiotherapy is managing sources of error that can affect the accuracy of radiation delivery. Among these, positional errors are a major concern, as small deviations in patient positioning can lead to misalignment between the radiation beam and target, reducing treatment efficacy and increasing risk of toxicity. Patient movement, setup errors, and organ motion are common factors that contribute to these uncertainties [5–7].

In modern radiation therapy, image guidance plays a vital role in ensuring accurate patient positioning and delivering high-precision treatments. The development of image-guided radiation therapy (IGRT) has significantly enhanced the accuracy of radiotherapy by integrating advanced imaging systems directly into treatment workflows. This integration allows for real-time verification and correction of patient positioning before radiation is delivered, ensuring the tumor is accurately targeted [8–10].

Linear accelerators (LINACs) utilize various imaging modalities to achieve precise patient positioning. Commonly used systems such as Varian TrueBeam, Elekta Versa HD, and Siemens Artiste incorporate imaging techniques such as cone beam computed tomography (CBCT), kV/MV imaging, and 2D orthogonal X-rays [11–13]. Each modality offers specific advantages, with CBCT providing high-quality 3D volumetric images that are essential for visualizing soft tissues and bony landmarks. These systems enable the radiation oncology team to perform imaging on a daily, weekly, or as-needed basis to verify patient positioning, in accordance with the specific requirements of the clinical protocol [14–17].

The TrueBeam system (Varian Medical System, Palo Alto, CA, USA) has been widely adopted for its robust imaging capabilities, including MV/kV imaging, CBCT, and 4D imaging, allowing clinicians to minimize positioning errors through automated image registration and advanced couch correction tools [18,19]. The Versa HD (Elekta AB, Stockholm, Sweden) similarly integrates CBCT along with the HexaPOD™ system [20,21], which provides both translational and rotational corrections, enhancing its precision in complex treatments such as stereotactic body radiotherapy (SBRT). The Halcyon system (Varian Medical System, Palo Alto, CA, USA) represents a significant advance in IGRT by simplifying and automating many of the positioning tasks [22,23]. Designed to optimize radiotherapy delivery, Halcyon mandates daily CBCT imaging for every treatment session, ensuring precise positioning through fast, accurate, and automated verification processes [24]. Unlike other systems that offer flexibility in imaging frequency, Halcyon’s emphasis on mandatory daily CBCT allows for consistent and reliable adjustments of translational shifts in the *x*, *y*, and *z* directions before each radiation fraction. Halcyon integrates a powerful dual-layer multileaf collimator and flattening filter-free technology [25], coupled with high-speed CBCT acquisition, which can capture volumetric images in ~15 s [25]. This rapid imaging capability, combined with a streamlined workflow, allows for efficient patient setup and real-time position correction, ultimately leading to higher throughput without compromising treatment accuracy.

Other LINAC systems, such as the Varian TrueBeam and Elekta Versa HD, offer flexible imaging protocols. While they are capable of performing daily CBCT, many treatment protocols use CBCT on a weekly or fractionated basis, depending on clinical needs and the complexity of the treatment plan. This flexibility allows clinicians to adjust the frequency of imaging based on factors such as tumor location, patient condition, and treatment modality. However, the lack of mandatory daily imaging in these systems can occasionally lead to minor misalignments or variations in treatment accuracy.

In this study, we aim to evaluate the efficacy of the daily CBCT imaging protocol in the Halcyon system at University Medical Shing Mark Hospital [26,27], focusing on its ability to detect and correct patient misalignments across a range of cancer types and treatment sites. By quantifying the translational shifts and necessary couch corrections, we seek to demonstrate the impact of daily CBCT on patient positioning and treatment accuracy in the Halcyon system.

## Materials and methods

### Patient selection

This retrospective study was conducted on 411 patients who received radiotherapy using the Varian Halcyon linear accelerator at the Oncology Department of ShingMark University Hospital between August 2022 and August 2024. The patients were divided into three groups based on tumor location: Head and Neck group (118 patients), Chest group (188 patients), and Pelvic group (105 patients). Patients were treated for a variety of cancers requiring precise radiation delivery to their respective tumor sites. All patients received their full course of radiotherapy using the Halcyon system, and daily CBCT imaging was performed for each treatment session.

For immobilization, patients were positioned using Qfix^®^ devices (Avondale, AZ, USA), including a thermoplastic S-frame mask for Head and Neck, an ArmsShuttle™ Elite Wing Board for the Chest, and a Carbon Fiber Pelvis System for the Pelvis.

### Imaging and treatment planning

For all patients, a CBCT scan was performed before each treatment session to verify patient positioning. The reference planning CT was obtained using a Siemens 16-slice CT scanner (model SOMATOM Scope, Siemens Healthineers, USA) [28] and was used for comparison to detect translational shifts in the *x*, *y*, and *z* directions. The treatment plans were generated using the Eclipse treatment planning system (TPS) based on the specific clinical requirements for each group.

For each patient, pre-treatment CBCT imaging was performed before each session using the Halcyon system's onboard imaging technology. The acquired CBCT images were subsequently registered against the reference planning CT scans, enabling the detection of translational shifts in patient positioning along the *x*, *y*, and *z* axes. Positional verification was performed using CBCT-based bone-matching, followed by soft tissue review. These shifts were quantified, and necessary adjustments were made using the automated couch correction feature to ensure correct alignment prior to treatment delivery. Treatment plans were generated using the TPS based on the patient’s CT. Patients received either volumetric-modulated arc therapy (VMAT) or intensity-modulated radiation therapy (IMRT), depending on the complexity of the case. The prescribed radiation dose and number of fractions varied with group and tumor type.

### Cone beam computed tomography imaging and positional verification

The Halcyon system utilizes daily CBCT imaging to verify and correct patient positioning before each treatment fraction. The steps involved in the CBCT process include:

CBCT image acquisition: A high-speed CBCT scan is captured in ~15 s before every treatment session.

Image registration: The acquired CBCT is automatically registered with the reference planning CT to identify positional discrepancies (Δ) in the *x*, *y*, and *z* directions [Δ*x* (left–right), Δ*y* (superior–inferior), Δ*z* (anterior–posterior)].

Couch adjustments: If translational shifts are detected, the treatment couch is adjusted manually or automatically to correct the patient position.

Positional verification: In certain cases, a second CBCT is performed to confirm proper alignment after couch adjustments.

The criteria for patient positioning were based on the following tolerances for translational shifts, determined by comparing CBCT images with the reference planning CT scans. Positional shifts were evaluated as follows:

Δ < 2.5 mm: Treatment delivered as planned.

Δ between 2.5 and 5 mm: Adjustments made to the patient position before treatment, but a repeat CBCT not required.

Δ > 5 mm: Significant adjustments to the treatment table or patient position are made, and a repeat CBCT performed to verify alignment before proceeding with treatment.

### Data collection and analysis

Positional shifts in the *x*, *y*, and *z* directions were collected for each patient across all treatment fractions. The magnitude of these shifts was analyzed using descriptive statistics, including the calculation of mean, standard deviation, and percentile values. For each treatment site (Head and Neck, Chest, Pelvic), histograms were generated to represent the distribution of positional shifts, and density plots were used to facilitate comparative analysis across treatment sites.

## Results

**Figure 1:**
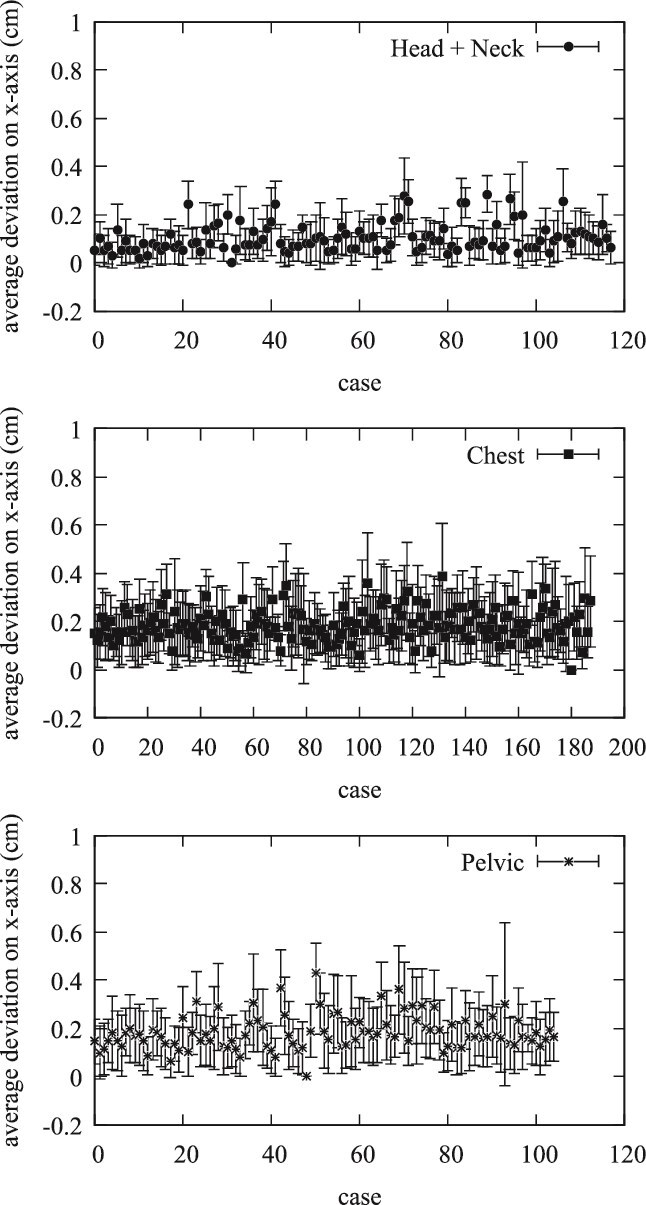
Average positional shifts on X-axis for Head and Neck, Chest, and Pelvic groups with standard deviations. This figure shows the average deviation in the x-axis for three treatment groups (Head and Neck, Chest, and Pelvis). Each panel represents the individual cases in the respective groups, with error bars indicating the standard deviation of the positional shifts.

The results of our study on the effectiveness of CBCT for patient positioning are illustrated through various analyses of positional shifts across three treatment groups: Head and Neck, Chest, and Pelvis. The average positional shifts on the *x*-axis, as shown in Fig. 1, indicated that the Head and Neck group exhibited a mean deviation of about 0.112 cm. In comparison, the Chest group demonstrated an average shift of 0.194 cm, while the Pelvic group recorded a mean deviation of 0.194 cm. The error bars highlight the variability within each treatment group, with the Head and Neck group displaying the smallest standard deviation, suggesting more consistent positioning.

**Figure 2:**
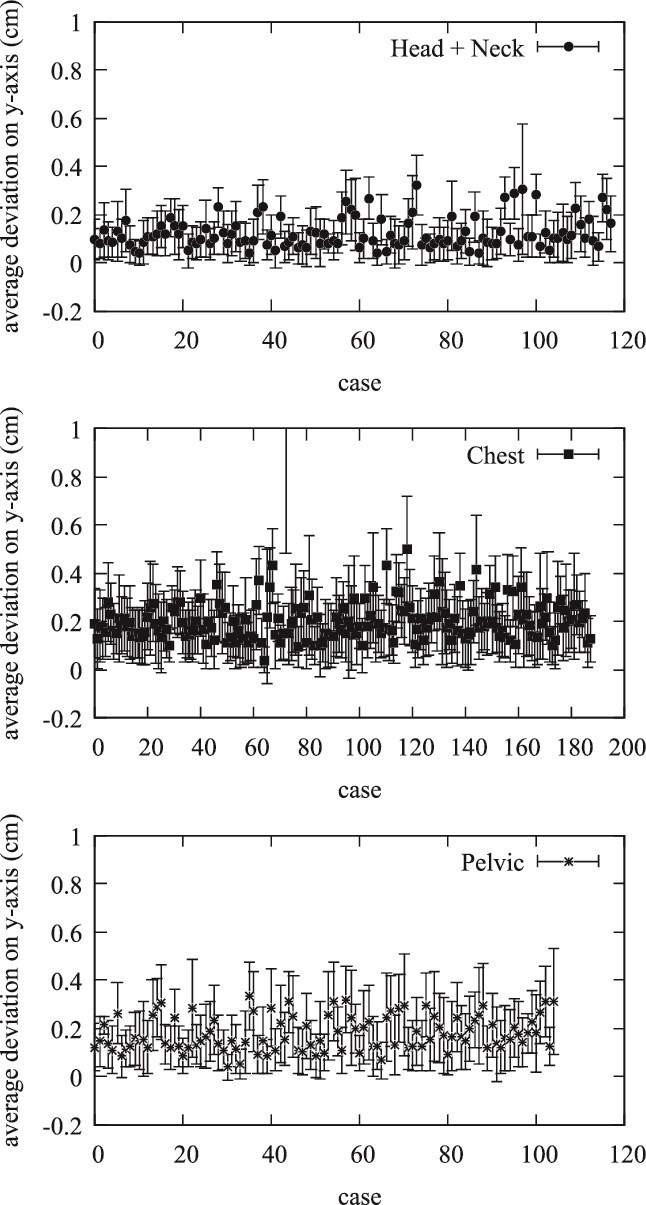
Average positional shifts on Y-axis for Head and Neck, Chest, and Pelvic groups with standard deviations. This figure illustrates the average deviation in the y-axis for three treatment groups (Head and Neck, Chest, and Pelvis), with error bars representing the standard deviation of the positional shifts in each case.

**Figure 3:**
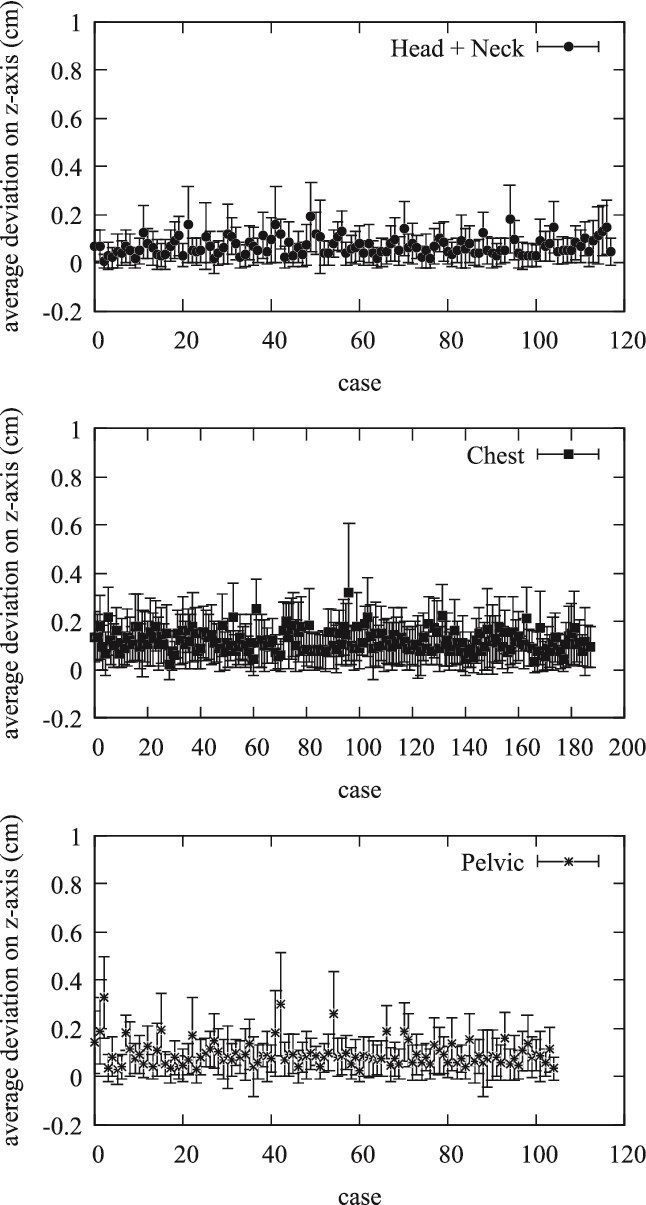
Average positional shifts on Z-axis for Head and Neck, Chest, and Pelvic groups with standard deviations. This figure illustrates the average deviation in the z-axis for the three treatment groups (Head and Neck, Chest, and Pelvis), with error bars representing the standard deviation for each case.

In terms of the *y*-axis, Fig. 2 reveals that the Head and Neck group had an associated average deviation of 0.134 cm. The Chest group recorded an average shift of 0.206 cm, while the Pelvic group indicated a mean shift of 0.195 cm. Consistent with the *x*-axis results, the Head and Neck group showed the least variability, reinforcing the precision of positioning within this cohort. The analysis of positional shifts on the *z*-axis, depicted in Fig. 3, further supports these findings. The Head and Neck group had a mean deviation of 0.07 cm, compared to 0.11 cm for the Chest group, and 0.085 cm for the Pelvic group. Once again, the error bars illustrate the range of positional shifts, with the Head and Neck group demonstrating the most reliable positioning.

**Figure 4:**
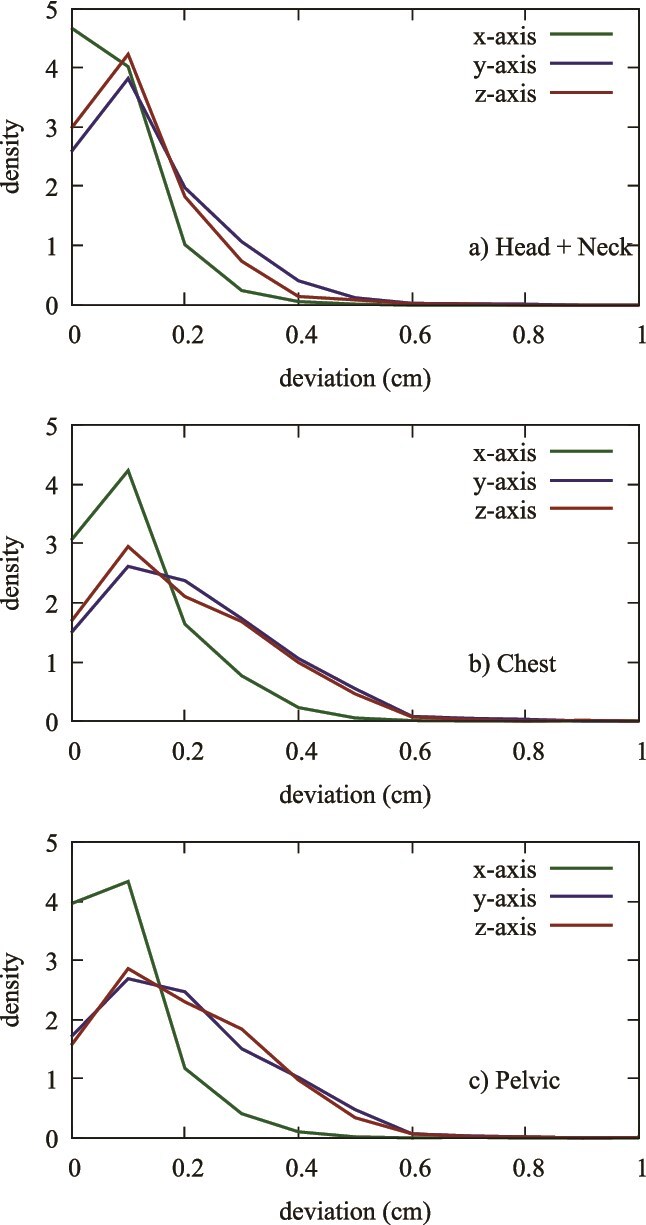
Normalized density distributions of positional shifts in x (a), y (b), and z (c) axes for Head and Neck, Chest, and Pelvic groups. This figure compares the normalized density distributions of positional shifts across the x-axis, y-axis, and z-axis between the Head and Neck, Chest, and Pelvic treatment groups.

**Figure 5:**
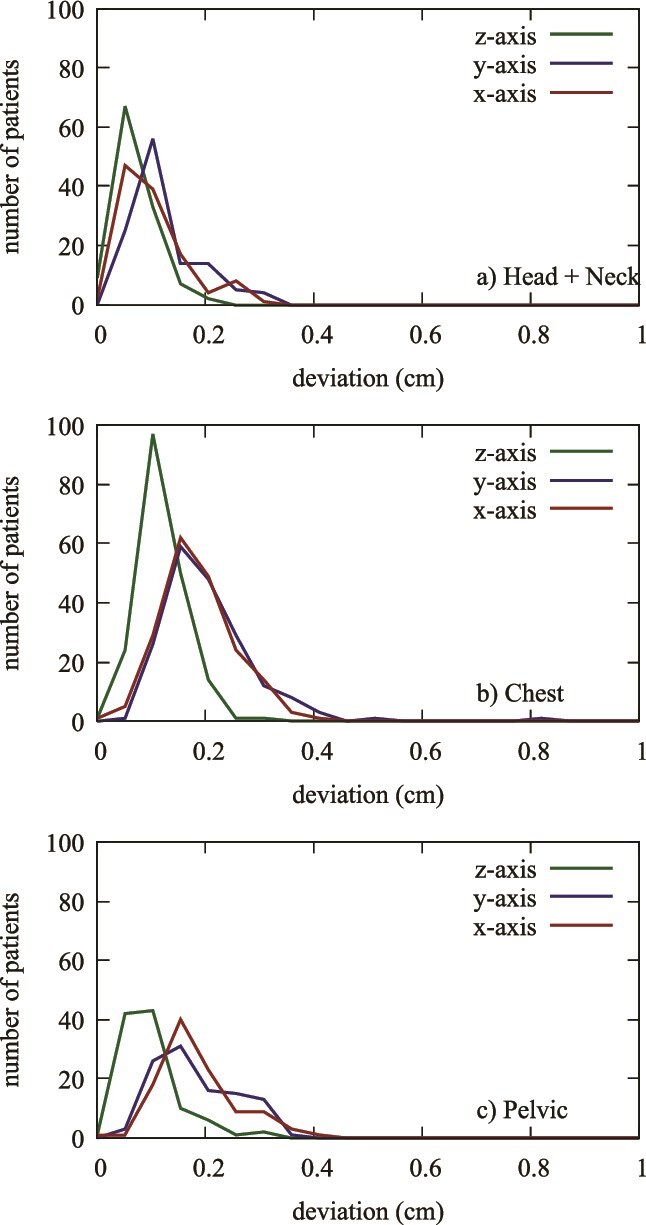
Distribution of positional shifts in x, y, and z axes for a single group (Head and Neck (a), Chest (b), and Pelvis (c)).

The normalized density distributions of positional shifts across the *x*-, *y*-, and *z*-axis are presented in Fig. 4. These distributions reveal distinct patterns for each treatment group, highlighting that positional shifts are most concentrated around the mean for the Head and Neck group, indicating a greater degree of consistency. In contrast, the Chest and Pelvis groups exhibited broader distributions, suggesting greater variability in positional shifts across all axes. In addition, Fig. 5 illustrates the distribution of positional shifts in the *x*, *y*, and *z* axes for each individual treatment group. Each panel shows the range of positional shifts within each group, with the Head and Neck group once again showing tighter distributions compared to the Chest and Pelvic groups, which displayed more scattered results.

Table 1 summarizes the positional shifts (Δ*x*, Δ*y*, Δ*z*) for the Head and Neck, Chest, and Pelvic groups. The data shows that the mean positional shifts are minimal, with the majority of shifts being <0.5 cm. Notably, the Head and Neck group exhibited the least deviations, particularly in the *y* and *z* axes, compared to the Chest and Pelvic groups. This indicates greater precision in patient positioning for treatments involving the Head and Neck region.

**Table 1:** Summary of positional shifts (Δ*x*, Δ*y*, Δ*z*) for Head and Neck, Chest, and Pelvic groups.

	Head and Neck			Chest			Pelvic		
	*z*	*y*	*x*	*z*	*y*	*x*	*z*	*y*	*x*
Mean	0.1	0.1	0.1	0.1	0.2	0.2	0.1	0.2	0.2
Min	0.0	0.0	0.0	0.0	0.0	0.0	0.0	0.0	0.0
Max	0.5	0.8	0.7	0.6	0.9	0.9	0.7	1.0	1.0
Percentile 1	0.0	0.0	0.0	0.0	0.0	0.0	0.0	0.0	0.0
Percentile 5	0.0	0.0	0.0	0.0	0.0	0.0	0.0	0.0	0.0
Percentile 25	0.0	0.0	0.0	0.0	0.1	0.1	0.0	0.1	0.1
Percentile 75	0.1	0.2	0.2	0.2	0.3	0.3	0.1	0.3	0.3
Percentile 95	0.2	0.4	0.3	0.3	0.5	0.5	0.3	0.5	0.4
Percentile 99	0.3	0.5	0.4	0.4	0.6	0.6	0.4	0.6	0.6

This table presents the mean, minimum, maximum, and various percentiles (1st, 5th, 25th, 75th, 95th, and 99th) of positional shifts in the *x*, *y*, and *z* axes for the head and neck, chest, and pelvic treatment groups. The data highlight the distribution and range of positional shifts, providing insights into the accuracy and variability of patient positioning in the halcyon system.

^a^Percentile *xx* means *xx*% of data is less than the given value. For example, a percentile of 75 = 0.1 indicates that 75% of the positional shifts were less than or equal to 0.1 cm. The percentiles thus offer a statistical overview of how positional shifts are distributed across the patient groups.

## Discussion

The evaluation of daily CBCT in the Halcyon system provides a comprehensive analysis of patient positioning accuracy across different treatment sites: Head and Neck, Chest, and Pelvic regions. This study focuses on the average positional shifts (Δx, Δy, Δz), their corresponding standard deviations, comparisons within and between treatment groups, and the implications of these findings. The average values of positional shifts and their standard deviations for all patients are summarized in Figs 1–3. The mean positional shifts are relatively small, with Δx, Δy, and Δz values around 0.1 cm for the Head and Neck group, while the Chest and Pelvic groups exhibit slightly higher deviations. The low standard deviations indicate consistent accuracy in patient positioning across all groups, suggesting that the Halcyon system effectively minimizes variability.

In a study assessing the effectiveness of CBCT for patient positioning accuracy, various significant results were reported [29,30], showing that CBCT imaging plays a critical role in maintaining high precision during radiation treatment. CBCT enables precise corrections in patient alignment, thereby reducing errors in positioning before radiation delivery. For example, a study by Flores-Martinez et al. [29] revealed that using CBCT with Surface Guided Radiation Therapy (SGRT) improved patient setup accuracy, reducing residual rotational errors significantly during treatments on Halcyon LINACs. These results underscore that CBCT imaging, especially when combined with other guidance systems, is highly effective in enhancing patient positioning precision, ensuring better outcomes for patients undergoing radiotherapy. Additionally, the paper by Huang et al. [30] entitled ‘Pediatric Cone Beam CT on Varian Halcyon and TrueBeam Radiotherapy Systems: Radiation Dose and Positioning Accuracy Evaluations,’ provides a detailed comparison between kV CBCT on the TrueBeam system and MV CBCT on the Halcyon system in terms of both radiation dose and positioning accuracy. The study emphasizes the importance of balancing imaging dose and positioning precision, particularly in pediatric patients who are more vulnerable to radiation risks due to their higher biological susceptibility and longer life expectancy. The authors found that the Halcyon system, with its MV CBCT imaging mode, demonstrated comparable or better positioning accuracy than the TrueBeam system, with reduced imaging dose when using "Low Dose" protocols.

Within the same treatment groups, a distinct pattern emerges: Δz < Δy < Δx. This trend, evident in Fig. 5, shows that deviations along the *z*-axis are the smallest, followed by slightly greater deviations in the *y*-axis, and the largest along the *x*-axis. This systematic difference in positional accuracy across the axes is consistent across all treatment groups, highlighting the importance of considering axis-specific adjustments in patient positioning protocols. When comparing positional shifts between the three treatment groups, intriguing insights arise. The deviations for Δz are similar across the Head and Neck, Chest, and Pelvic groups, indicating uniform accuracy along this axis. However, for Δy and Δx, the Head and Neck group demonstrates significantly smaller deviations compared to the Chest and Pelvic groups. This finding suggests that the Halcyon system achieves higher precision in the Head and Neck region, which is particularly critical for treatments involving sensitive structures such as the brain and surrounding organs. The observed differences in patient positioning accuracy across treatment groups may be attributed to the type of immobilization devices used. The Head and Neck group demonstrated the highest positioning accuracy, likely due to the use of a thermoplastic S-frame mask, which provides rigid immobilization and minimizes setup errors. In contrast, the Chest group, where patients were positioned using a Qfix® ArmsShuttle™ Elite Wing Board, showed slightly greater variability, possibly due to respiratory motion and less rigid immobilization. The Pelvic group, which utilized the Qfix® Carbon Fiber Pelvis System, also exhibited higher positional deviations, likely influenced by natural organ motion and pelvic repositioning between sessions. These findings emphasize the importance of patient immobilization techniques in achieving optimal setup accuracy and reducing positional uncertainties in radiotherapy.

The quantitative data provide a detailed breakdown of positional uncertainties, revealing that the maximum observed positional shift is 1 cm, which occurred in <2% of cases. Notably, 98% of positional shifts were <0.5 cm, and 75% were below 0.2 cm. This high level of precision is crucial for effective radiation therapy, as it minimizes the risk of targeting errors and ensures optimal treatment delivery. Specific treatment group analyses reveal unique patterns. The Head and Neck group, which includes cases such as brain and tongue cancers, showed minimal positional shifts with tight distributions around the mean values, indicating high precision. The Chest group, covering breast, lung, and esophageal cancers, exhibited slightly larger shifts, particularly along the *y*- and *x*-axis. In contrast, the Pelvic group, which includes cancers such as rectal and prostate, showed the largest deviations, especially along the *x*-axis, suggesting more variability in positioning. Figures 1–5 collectively illustrate these findings. Figure 3 depicts the average deviation on the *z*-axis for the three treatment groups, showing small deviations with low variance. Figure 2 illustrates the average deviation on the *y*-axis, following a similar trend to the *x*-axis but with slightly larger deviations, particularly in the Pelvic group. Figure 1 displays the average deviation on the *x*-axis, highlighting the largest deviations in the Pelvic group while the Head and Neck deviations remain minimal. Figure 4 presents density distributions across the *x*, *y*, and *z* axes, showing that the Pelvic group has greater density around larger deviations, whereas the Head and Neck group demonstrates a tighter distribution. Lastly, Fig. 5 shows histograms for deviations across all treatment sites, indicating that most corrections fall within 2–3 mm, though some larger deviations necessitated additional adjustments. Table 1 provides a detailed breakdown of positional shifts (Δx, Δy, Δz) for the Head and Neck, Chest, and Pelvic groups. The data reveals that the mean positional shifts are minimal across all groups, with the majority of shifts being <0.5 cm. Specifically, the Head and Neck group exhibits the smallest deviations, particularly along the *y* and z axes, compared to the Chest and Pelvic groups. This indicates greater precision in patient positioning for treatments involving the Head and Neck region, which is critical for targeting sensitive anatomical structures.

Table 1 also highlights the maximum positional shift observed, which can reach up to 1 cm, though this is rare, occurring in <2% of cases. Notably, 98% of the positional shifts are <0.5 cm, and 75% are <0.2 cm. These findings underscore the high level of accuracy achieved by the Halcyon system, ensuring that patient positioning remains within clinically acceptable limits and thereby enhancing the effectiveness of radiation therapy.

The findings from this study suggest that the Halcyon system may be highly effective in enhancing patient positioning accuracy. The low mean positional shifts and narrow density distributions suggest that the system can reliably position patients with minimal deviations. This is particularly important for treatments requiring high precision, such as those for the Head and Neck region, where even minor inaccuracies may significantly impact treatment outcomes. While the results are promising, it is important to acknowledge the limitations of this study. The sample size for each treatment group may affect the generalizability of the findings. Future research should aim to include larger cohorts and explore the impact of different patient demographics on positioning accuracy. Additionally, investigating the integration of advanced imaging techniques and adaptive radiotherapy protocols will enhance the precision of patient positioning, ultimately leading to improved patient outcomes in radiation therapy.

## Conclusion

This study suggests that the Halcyon system has the potential to enhance patient positioning accuracy through daily CBCT. The analysis of positional shifts across the Head and Neck, Chest, and Pelvic treatment groups reveals that the system maintains high precision with minimal deviations. The majority of positional shifts are <0.5 cm, with 75% being under 0.2 cm, underscoring the reliability of the Halcyon system in clinical settings. The findings highlight the system’s capability to deliver consistent and accurate patient positioning, which is crucial for the efficacy of radiation therapy. The low mean positional shifts and narrow density distributions across all axes indicate that the Halcyon system can effectively minimize targeting errors, thereby enhancing treatment outcomes. Future research should focus on expanding the sample size and exploring the impact of various patient demographics on positioning accuracy. Additionally, integrating advanced imaging techniques and adaptive radiotherapy protocols could further improve the precision and effectiveness of patient positioning. In conclusion, the Halcyon system may represents a significant advancement in radiation therapy, providing a robust solution for accurate patient positioning and contributing to improved clinical outcomes.

## Data Availability

Research data are stored in an institutional repository and will be shared upon request to the corresponding author.
